# Artificial Intelligence (AI) and Cardiovascular Diseases: An Unexpected Alliance

**DOI:** 10.1155/2020/4972346

**Published:** 2020-06-27

**Authors:** Silvia Romiti, Mattia Vinciguerra, Wael Saade, Iñaki Anso Cortajarena, Ernesto Greco

**Affiliations:** ^1^Department of Clinical Internal Medicine Anaesthesiology and Cardiovascular Sciences, Sapienza University of Rome, Rome, Italy; ^2^MBA IESE, Financial Advisory M&A Transaction Serv. Deloitte, Barcelona, Spain

## Abstract

Cardiovascular disease (CVD), despite the significant advances in the diagnosis and treatments, still represents the leading cause of morbidity and mortality worldwide. In order to improve and optimize CVD outcomes, artificial intelligence techniques have the potential to radically change the way we practice cardiology, especially in imaging, offering us novel tools to interpret data and make clinical decisions. AI techniques such as machine learning and deep learning can also improve medical knowledge due to the increase of the volume and complexity of the data, unlocking clinically relevant information. Likewise, the use of emerging communication and information technologies is becoming pivotal to create a pervasive healthcare service through which elderly and chronic disease patients can receive medical care at their home, reducing hospitalizations and improving quality of life. The aim of this review is to describe the contemporary state of artificial intelligence and digital health applied to cardiovascular medicine as well as to provide physicians with their potential not only in cardiac imaging but most of all in clinical practice.

## 1. Introduction

According to the fifth edition of the European Cardiovascular Disease Statistics (published in 2017 by the European Heart Network (EHN)), cardiovascular diseases (CVD) represent the leading cause of death and morbidity in Europe. In 2015, over 85 million people were affected by CVD (48% men and 52% women) in the continent, leading to 3.9 million deaths (45% of all causes of death). In the European Union (EU), 49 million people were dealing with CVD, out of which over 1.8 million resulted in death (European Cardiovascular Disease Statistics 2017).

CVD represent a significant economic cost for society, around $351.2 billion in US, chronically affecting patients' quality of life [1]. The EU has estimated that the overall yearly cost amounts to €210 billion, allocating around 53% to healthcare costs (€111 billion), with 26% related to productivity losses (€54 billion), and the remaining 21% (€45 billion) to the informal care of people with CVD (European Cardiovascular Disease Statistics 2017).

In 2017, in Italy, 4.4 patients per every thousand inhabitants suffer from some kind of cardiovascular disease and 232 992 people died from it (Istat data).

The incidence of disability in those who survived was very high, chronically impacting patients' quality of life and healthcare costs. The Italian pharmaceutical industry uses 23.5% of its fund expenditures for CVD treatment drugs (https://www.epicentro.iss.it).

This data supports the fact that CVD, despite the significant advances that occurred in the diagnosis and treatments, are still the most common cause of morbidity and mortality in Europe.

Early accurate diagnosis and prognosis evaluation are key to improve and optimize CVD outcomes.

Artificial intelligence (AI) techniques such as machine learning (ML), deep learning (DL), and cognitive computer can play a critical role in the early detection and diagnosis of CVD, as well as outcome prediction and prognosis evaluation. Widespread data acquisitions of electronic health records (EHRs) have generated massive datasets (quantitative, qualitative, and transactional data) that require AI techniques to be interpreted [2].

AI techniques can also assist physicians to make better clinical decisions enabling early detection of subclinical organ dysfunction, through the use of clinically relevant information that can be found in the massive amount of data and, thus, improving quality and efficiency of healthcare delivery [3].

Telemedicine and mobile health (mHealth) are also becoming important in the prevention of CVD and general improvement of healthcare [4–6]. Likewise, Internet of Things (IoT) can be a radical game changer in heart disease healthcare environment; patients' acquired data can be sent to remote physicians who will be able to constantly know patients' physical status in real time [7, 8]. The aim of this review is to describe the contemporary status of artificial intelligence applied to cardiovascular medicine and its potential to change the way of how we generate knowledge, interpret data, and make decisions.

## 2. What Is Artificial Intelligence?

Artificial intelligence (AI) is a computer system able to perform tasks that ordinarily require human intelligence such as receiving perceptions from the environment and performing actions using algorithms, heuristics, pattern matchings, rules, deep learning, and cognitive computing. A group of pioneers first coined the term in 1956 at Dartmouth College in New Hampshire, USA. In 1958, Rosenblatt [9] developed the first precursor to current neural networks: the perceptron, a “brain model” for supervised learning of binary classifiers. In 1986, Rymelhart et al. [10] described a new learning procedure, backpropagation, for networks of neurone-like units able to learn any function. Although big progress was made through the'90s and 2000s, only in 2012, when Krizhevsky and colleagues won the ImageNet ILSVRC contest with a deep convolutional neural network to classify objects using GPUs (graphics processing units) to accelerate network training, an explosion of research activity in the neural network field started to happen [11, 12].

The continuous development of AI techniques, mainly in the subdomains of ML and DL, has quickly attracted the attention of clinicians to create new integrated, reliable, and efficient methods for providing quality healthcare.

Imaging is the focus of interest and research when it comes to AI in cardiovascular medicine.

The advantages of using ML models in echocardiography lie in the reduction of inter- and intraoperator variability as well as in the provision of additional predictive information that may be too subtle to be detected by human eyes [13–15].

Another interesting potential application of AI techniques could be in cardiac CT, for patients with suspected CAD. For patients suffering from these conditions, the association between cardiac CT and ML algorithms has shown a potential in clinical practice to take noninvasive approaches and to detect functional information beyond atherosclerotic plaque characterization [16–18].

Besides diagnostic imaging, another interesting application of ML in cardiology could be in the automatic detection of anomalies in electrocardiograms.

### 2.1. Machine Learning

Machine learning (ML) is a subfield of AI intended to “teach” computers to analyze vast datasets in a quick, accurate, and efficient way, through the use of complex computing and statistical algorithms [13].

These algorithms are able to identify patterns on new data that match with existing data they already “learned from” and make predictions based on them [19].

In ML, an input **x** and an output **y** follow a functional relationship *y* = *f* (**x**), called the predictive model [19].

ML can be classified into three groups based on the way the predictive model learns and accumulates data [20–24].

1. Supervised learning (e.g., logistic regression, SVM, and neural networks): uses human labelled datasets, generally used to develop models that predict or classify future events or find the most relevant variables to the outcome. Both **x** and ***y*** are known and the predictive model improves and benefits from data training.
2. Unsupervised learning (e.g., cluster analysis): the software is capable of finding hidden structures in datasets, without prior categorization of the training set (only **x** is known). This has the potential to identify novel relationships within the data.
3. Reinforcement learning: reward-based learning (typically used in gaming and robotic applications), based on interactions with an environment in which positive and negative reinforcements contribute to the improvement of the predictive model. It requires the machine to be equipped with systems and tools that can not only improve its learning but also understand the characteristics of the surrounding environment, such as sensors, cameras, and GPS.

### 2.2. Deep Learning

Deep learning (DL) is a supervised ML technique that uses neural networks and is characterized by automated algorithms that are able to extract meaningful patterns from data collections [4]. It mimics the complexity of a human brain, being able to learn complex hierarchical representations from data that has multiple levels of abstraction [4, 25]. The programmer enters known data into the machine in a way that allows algorithms to respond correctly even when faced with fully new data. The neural network learns through experience, reads data, builds hierarchical architectures, and provides advanced input-output levels. It can capture complex nonlinear relationships between input-output outcome variables. The average error of outcomes and their predictions can be minimized by estimating the weights of input and outcome data [3].

Physicians diagnose based on their knowledge, experience, and cultural background. Deep learning could be very successful at this point, broadening and improving medical knowledge, particularly for nonexpert physicians.

DL can explore more complex nonlinear patterns in the data than classic neural networks by using more hidden layers. For this reason, the application of DL in the medical research field has recently become popular due to the increase in volume and complexity of data, particularly for the imaging analysis area [3, 26].

DL is also playing a prominent role in Facebook's image recognition program, speech recognition in Apple's Siri and Amazon's Alexa, Google brain and robots, etc. [27].

In the medical context, the most widespread deep learning algorithms are convolution neural networks (CNN), recurrent neural networks, deep belief networks, and deep neural networks.

## 3. Electronic Health: Mobile Health and IoT

Electronic health (eHealth), or digital health, refers to the use of emerging communication and information technologies, basically the Internet, and aims to improve health and healthcare [5].

### 3.1. Mobile Health

Mobile health (mHealth) is a subfield of eHealth, characterized by the use of mobile and wireless technologies to improve healthcare.

When it comes to the prevention of CVD, mobile devices are a great promise especially thanks to dietary self-monitoring apps, physical activity monitors, and blood pressure (BP) monitors. These applications can help patients achieve a healthful weight, improve physical activity, quit smoking, control blood glucose, and manage BP and lipids to achieve target levels [5, 28–32].

Apple, in collaboration with Stanford Medicine, successfully conducted a research study [33] to evaluate whether the Apple heart Study App (a mobile medical app that analyzes pulse rate data) could use data collected on the Apple Watch to identify irregular heart rhythms (atrial fibrillation and other arrhythmias). This study paved the way to a novel large-scale pragmatic study, in which outcomes and findings can be reliably assessed with user-owned devices.

mHealth became also important in cardiovascular medicine with the development of portable computer devices and miniaturized cardiac imaging devices. Moreover, “big data” from mHealth and telemedicine can be integrated with AI techniques, helping cardiologists make better clinical decisions [4, 34, 35].

### 3.2. Internet of Things

The Internet of Things (IoT) is the network of physical objects, devices, vehicles, buildings, and other items that are embedded with electronics, software, sensors, and network connectivity, that enables the collection and exchange data [8].

The general architecture of IoT medical applications consists of three layers: the sensing layer composed of sensors worn or carried by patients, the transport layer composed of connecters, and the application layer composed of remote server [7]. Thanks to these, acquired data is transmitted to a remote server and saved in a database or displayed in real time by physicians.

It is especially useful for elderly and chronic disease patients, shifting healthcare from a passive activity into a pervasive one. In cardiovascular medicine, physicians are able to monitor patients in real time thank to the collection of data such as blood pressure, ECG, and SpO_2_, being aware of patient's health conditions and diagnosing or forecasting dangerous events. This will radically change patient's quality of life: they will be able to lead a more normal life, receiving medical care at their homes and thus reducing hospital visit frequencies. When IoT techniques are combined with real-time analytical algorithms, they can also become a mean to warn about potential attacks in advance.

In the heart failure care field, wearable sensors coupled with ML analytics can be potentially used to improve clinical outcomes and reduce hospitalizations [36–38]. Heart failure is a chronic disease with acute exacerbations that reports high rates of hospitalization and mortality year after year (one-year hospital readmission rate of more than 50%, and one-year mortality rates of 30%) [39] and involves a worldwide expenditure of around $31 billion [40] yearly. It is estimated to affect over 26 million adults worldwide [41], 6.5 million only in the United States [42], 14 million in Europe, and 1 million in Italy, with an increasing prevalence rate related to aging (over 10% of the patients are 70 years old or older) [43]. It is the leading cause of hospitalization among people over the age of 65 [44] in Italy, with over 190,000 hospitalizations every year and an expenditure of at least €500 million [45] with 9.4 days of hospital stay on average [45]. Due to the fact that the patient care cost increases in relation to the severity of the disease, for NYHA IV patients, it is 8 to 30 times higher than that for NYHA II patients [46].

Despite the big progresses in therapies and prevention techniques, the incidence of rehospitalization is of 25% in a 1-month period and increases to c.50% in a 6-month [46] period, with the mortality rate still being c.50% in a 5-year period after diagnosis [47].

Due to the high cost of hospitalizations (the average length is 5–10 days) [41] and the high rates of morbidity and mortality (especially between the elderly population), the potential of IoT-based devices stands out. In the LINK-HF study [36], it was demonstrated that machine learning models that use data from VitalPatch®, a wearable sensor, can more accurately forecast heart failure exacerbation than invasive devices. The sensor layer used in the mentioned study was made up of a multisensory patch placed on the chest that recorded physiological data. Data was uploaded via smartphone (transport layer) to a cloud analytics platform (application layer) which used ML algorithms to analyze the collected data. Sensible Medical Innovations Ltd. (Netanya, Israel) developed ReDS™ [48], a wearable hemodynamic noninvasive technology able to detect the amount of lung fluid concentration. Comparing ReDS™ technology to high-resolution chest computed tomography (CT) in patients with acute heart failure resulted in ReDS™ being more suitable for the management of acute events recurrence in recently discharged patients [49].

With the aim to significantly improve heart failure patients' life, Vectorious has created V-LAP™. It is the first battery-less wireless microcomputer for cardiac monitoring, a novel Left Atrial Pressure monitoring system with a real-time tracking method based on AI that introduces remote heart failure care. By tracking trends of left atrial pressure readings with theV-LAP™, physicians can detect heart failure exacerbation before the onset of symptoms, change treatments, or modify doses of medication in order to reduce adverse complications (https://www.vectoriousmedtech.com).

Sievert et al. [50] successfully performed the first human experience with the V-LAP™. The V-LAP™ sensor was implanted using a transseptal access, with angiographic and echocardiographic guidance, and showed successful results in NYHA Class III patients regarding to implantation safety and feasibility. Other invasive device developed to manage HF hospitalizations was CardioMEMS™ HF System (Abbott, Lake Bluff, Illinois, United States): a permanent wireless pulmonary artery pressure (PAP) monitoring system. The device granted a big impact on the reduction of hospitalizations, as it allowed a tailored online management designed through the PAP data. The CHAMPION trial demonstrated effectiveness in the use of CardioMEMS™, reducing on average a 33% of the HF hospitalizations in NYHA Class III Heart Failure Patients over a follow-up period of 18 months [38].

## 4. Applications in Cardiovascular Imaging

AI techniques such as machine learning, deep learning, and cognitive computing have the potential to change the way in which cardiology and cardiovascular medicine are practiced (e.g., how we generate knowledge, interpret data, and make decisions), especially in cardiovascular imaging.

### 4.1. Echocardiography

The role of echocardiography is crucial in the diagnosis and management of cardiovascular diseases and accurate quantitative assessment of cardiac structures and functions. However, it still depends on the interoperator variability and experience. AI tools, in particular machine learning, provide new possibilities to enhance the accuracy of image interpretation in clinical echocardiography practice, especially between nonexpert clinicians. ML models trained to learn different features in an image are able to recognize a wide range of specific disease patterns, taking account of each pixel and their relationships [13].

ML models bring the potential to interpret, in an automated manner, the unused data that is generated by the advent of multidimensional imaging modalities (such as 3D echocardiography and speckle tracking) [13]. This leads to advantages in the reduction of analysis time and in the increase of reproducibility [4].

3D echocardiographic automated analysis can be performed thanks to HeartModel^A·I·^, a software package that uses a model-based algorithm. The algorithm that is integrated in the software is capable of automatically calculate the following in few seconds: (i) the volumes of the left chambers (atrium and ventricle), (ii) the systolic flow, and (iii) the ejection fraction of the LV from the data acquired with 3D echocardiographic techniques (https://www.ultrasound-heartmodel.it). In addition, the software also obtains simultaneously the atrium volume from the same eco-3D dataset, providing a more complete assessment of the function of the atrium, if we compare it to conventional measurement systems [51]. Another important advantage of this algorithm is that it has been designed to analyze eco-3D datasets acquired in single-beat mode. This can be particularly useful in patients whose 3D analysis is difficult, as could be the case of patients with frequent arrhythmias or those with breathing difficulties.

A wide range of cardiovascular diseases can benefit from ML models in clinical echocardiography practice.

Sengupta et al. [52] developed a cognitive machine-learning algorithm, trained with speckle tracking echocardiographic (STE) data, to differentiate constrictive pericarditis from restrictive cardiomyopathy. This study demonstrated the feasibility and effectiveness of a cognitive computing machine learning approach for automated interpretations of STE data.

Narula et al. [14] also showed that supervised learning algorithms could differentiate athlete heart and hypertrophic cardiomyopathy, using STE data, more accurately than traditional measure systems. Another potential field of application of ML models in echocardiography is heart valve disease (HVD) [53, 54]. HVD is an increasingly common pathology which can benefit from cardiac imaging ML integration through early diagnosis, treatment, or surgery planning [54]. Playford et al. [55] evaluated whether AI could impute the aortic valve area (AVA) in aortic valve stenosis from other echocardiographic data, without the need of measuring left ventricular outflow tract (LVOT); a high accuracy (0.95) was obtained. In order to assess mitral regurgitation severity, Moghaddasi and Nourian [56] introduced novel features to detect micropatterns out of echocardiography images. Their proposed method achieves a 99.38% sensitivity and 99.63% specificity rates in the detection of MR severity.

Ortiz et al. in 1995 [57] leaded the way to the application of AI tools in the field of heart failure (HF). They used a neural network method, based on echocardiographic data, to assess a one-year prognosis in a HF patient. Their work concluded that neural networks could more accurately predict outcomes than linear discriminant analysis (accuracy of 90% and sensitivity of 71.4% vs 67.4% and 67.5%, resp.).

Subsequent studies also showed that echocardiographic data and clinical factors can be used by AI tools to facilitate HF diagnosis, classification, severity estimation, and prediction of adverse events [21, 58–60], particularly in patients with preserved ejection fraction [61, 62]. Recently, Ouyang et al. [63] developed and performed successfully with an accuracy over 0.92 a novel video-based deep learning algorithm: EchoNet-Dynamic. This model, using 3D convolutional neural network, is able to assess from echocardiogram videos alone cardiac function (segmentation of left ventricle and estimation of ejection fraction) with an accuracy equal to or better than human experts.

### 4.2. Magnetic Resonance Imaging

In cardiac MRI, ventricular segmentation is one of the fields with more potential for the application of ML models. It makes it possible to quantify the volumetry and improve the efficiency and reproducibility of clinical assessments [64–66]. Avendi et al. [65] used deep learning algorithms (i.e., convolutional neural networks and stacked autoencoders) trained through cardiac MRI datasets, for the automatic detection and segmentation of right ventricular (RV) chamber foreseeing the accuracy of these algorithms. Likewise, for left ventricular segmentation, several automated neural networks have been successfully developed, especially for cardiac cine MRI [66–68]. Another application of ML in cardiac MRI takes place in the detection of subacute or chronic myocardial scar [69].

Dawes et al. [70] used supervised machine learning of 3D patterns of systolic cardiac motions, to predict (independent of conventional risk factors) adverse outcomes (early death or right heart failure) in patients with pulmonary diseases.

### 4.3. Cardiac Computed Tomography

ML image analysis techniques in cardiac CT are increasingly used in the diagnosis and risk assessment of coronary artery disease (CAD) and atherosclerosis (e.g., coronary artery calcium scoring and fractional flow estimation). Coronary computed tomographic angiography (CCTA) is a noninvasive modality to detect coronary artery disease. It generally overestimates stenosis severity compared to invasive angiography, and angiographic stenosis does not necessarily imply hemodynamic relevance when fractional flow reserve (FFR) is used as a reference [71]. Therefore, several ML models have been developed [17, 18, 71] to determine noninvasive FFR and improve the performance of CCTA by correctly reclassifying stenosis that are hemodynamically nonsignificant.

In order to characterize coronary plaque, automatic coronary artery calcium scoring in CCTA using ML models provide added clinical value by reducing false positive and interobserver variability [72, 73]. Wolterink et al. [73] used supervised machine learning to directly identify and quantify coronary artery calcification (CAC).

Gonzàles et al. [74] used convolutional neural network to calculate Agatston score from CT without prior segmentation of coronary artery calcification.

Another application of ML on cardiac CT is in the prognosis [75] and myocardial infarction detection, through the use of texture analysis methods [76].

Preliminary Results of the SMARTool Project [77] introduced a novel concept on the management of CAD patients (diagnosis, prognosis, and treatment) based on ML risk stratification and Computational Biomechanics. ML analysis was performed from retrospective and prospective data (clinical, biohumoral, CCTA imaging, lipidomics, etc.) in order to discriminate low- and medium-to-high risk patients. The CAD diagnosis module was based on the 3D reconstruction of the coronary arteries and the noninvasive estimation of smartFFR, whereas CAD predictions are based on complex plaque growth computational models.

### 4.4. Applications in Electrocardiography

Besides diagnostic imaging, electrocardiography is another field that could also benefit from the application of ML to its activities.

The electrocardiogram (ECG) is the most widely used tool to identify abnormalities in the electrical heart activity. ML models, especially the subfield DL, have enabled the automatic detection of anomalies in electrocardiograms, reducing the time of interpretation and the dependency on individual variability [78, 79].

Supervised learning algorithms have been largely developed to be applied on heart rhythm classification [80, 81].

The possibility to both train and test the different algorithms is provided by public databases such as MIT-BIH Arrhythmia from Physionet Project (https://physionet.org/physiobank/database/). In contrary, in the unsupervised learning analysis, the algorithms process datasets that lack a default categorization [82]. Data that has not previously been labelled is treated with this approach and afterwards grouped with different methods into ECG phenotypes subgroups [83, 84].

In particular, Lyon et al. [79], putting together data with similar structures, have identified and classified ECG phenotypes associated with arrhythmic risk markers in hypertrophic cardiomyopathy.

DL optimizes ECG interpretation performing patient stratification as documented by recent reports [85, 86]. Interestingly, Hannum et al. have trained a 34-layer deep neuronal network (DNN) as a DL model, to identify and classify 12 different types of arrhythmia. The obtained results are excellent compared to a dataset of recordings annotated by a certified board of cardiologist [85].

Similarly, in the study of Attia et al., they trained a 6-layer DNN to detect left ventricular systolic dysfunction through the performance of a stratification superior to B-type natriuretic peptide (BNP) screening blood tests [86].

## 5. Conclusion and Future Prospects

Physicians have both a big opportunity and responsibility to actively track the continuous developments of AI techniques and use and apply them according to their needs, in order to find concrete supporting tools for their clinical practices. The onset of artificial intelligence in the cardiovascular field is bringing wide possibilities also to provide new personalized cares. The way we practice cardiology, especially in the cardiac imaging field, is going to change and physicians need to be ready. mHealth and telemedicine are establishing new connections between patients and physicians, switching healthcare from a passive activity into a pervasive one. Physicians should not be afraid of the integration of AI into cardiology but should embrace it, since their expert knowledge will keep being vital under any circumstances.
